# Answer to Böhmert et al

**DOI:** 10.1186/s12940-020-00676-w

**Published:** 2020-11-25

**Authors:** Maël Dieudonné

**Affiliations:** 1 Max Weber Center, Institut des Sciences de l’Homme, 14 avenue Berthelot, F-69007 Lyon, France; 2 Health Services and Performance Research, University Hospital of Lyon, 162 avenue Lacassagne, F-69003 Lyon, France

**Keywords:** EMF health effects, Functional somatic syndromes, Health beliefs, IEI-EMF, Media effects, Modern health worries, Nocebo response, Risk perception

## Abstract

I thank Böhmert et al. for their commentary of my review, although their criticisms suggest a misunderstanding of its aims and scope. It does not discuss their comprehensive model per se, but as the latest formulation of a hypothesis that was put forward almost 15 years ago, and only as regards its ability to explain EHS symptoms as they are known to occur. While the authors reassert the strengths of their model, they do not properly address the limitations pointed out in my review, pertaining to: (1) the lack of proven explanations for the origins of beliefs in EMF harmfulness; (2) the realism of experimental studies of EHS; (3) the existence of situations contradicting predictions of their model. Thus, while it seems promising, its applicability to EHS remains to be properly demonstrated. A diversification of the methods used to study EHS seems the only way forward.

I thank the authors for their commentary. They represent a research team whose contribution to the study of EHS, arguably the most consistent in recent years, they wish to defend against seemingly misguided criticisms. However, their answer partly rests on a misunderstanding of the aims and scope of my review.

First, it does not analyse their comprehensive model of idiopathic environmental intolerance per se, but as the latest formulation of a hypothesis that was put forward almost 15 years ago to explain EHS symptoms [1, 2]. His proponents underlined the role of cognitive by opposition to biophysical mechanisms, drawing from the observation that perceived exposure was correlated with EHS persons’ reactions in experimental settings, whereas actual EMF exposure was not. The authors went further by developing a unified model of EHS and other environmental intolerances, using recent advances in the study of brain functioning. The strengths of their model, they reasserted eloquently in their commentary. But contrary to their assumption, it is not discussed in my review as the ambitious theoretical proposition that it is, but simply with regards to its ability to explain EHS symptoms, as they occur concretely among affected people.

In that prosaic perspective, the authors’ model still suffers from a significant weakness: the lack of proven explanations for the origins of beliefs in EMF harmfulness. Their commentary confirms that they usually take such beliefs for granted: see how often they write “*once the causal belief is established…*” (emphasis is mine). They do suggest various possibilities as to *how* that belief becomes established, while becoming noticeably less assertive. Indeed, what tangible evidence do they offer? How do they know that “*media reports, personal communication, or the observation of behaviours*” related to EMF are sufficient to “*change critical processes and the order of events*” in real life, ultimately leading to EHS? Can they provide any example involving actual EHS persons? It seems not.

Of course, the authors are not entirely without evidence. But the one they use is fragile, as emphasized in my review and elsewhere [3, 4]: it only comes from experimental studies whose settings bear little to no resemblance to the reality of EHS, all the more so when they concern other conditions with other environmental triggers. Great caution is thus necessary when extrapolating their results to EHS, as they do not prove that the phenomena they observe neither are responsible for EHS symptoms in real life, nor are specific to EHS or even to environmental intolerances as a whole.

The authors dismiss that issue by asserting that “*laboratory experiments are intended to demonstrate a mechanistic principle, not to create a patient.*” Arguably, as far as human subjects are concerned. But to demonstrate the responsibility of such “*mechanistic principles*” in actual conditions like EHS, experiments must also be proven to adequately reflect their clinical reality. Otherwise, all that remains are assumptions, no matter how elaborated. The authors seem to address on theoretical grounds what are ultimately empirical limitations, somehow mistaking conceptual strength for ecological validity. But if they wish to prove that their model adequately explains EHS symptoms, they should not avoid the discussion on the realism of their experiments. They should also not contend themselves with various possibilities regarding the establishment of beliefs in EMF harmfulness, which they deem plausible and compatible with their model: the burden falls on them to demonstrate unequivocally that these possibilities are true as regards EHS.

This may appear as quibbling. But these questions are crucial for the study of EHS, because there seems to be huge discrepancies between the exposure to negative information on the health effects of EMF, through whatever means conceivable, and the development of clinically significant EHS symptoms. In France, for instance, the largest TV audience ever reached by a report on EMF harmfulness was 1.82 million people, in May 2011. Today, the most regular source of negative coverage about EMF is a magazine from a whole food supermarket chain, which is circulated in more than 230,000 copies every month. However, the number of EHS persons remains in the low thousands, as can be judged by the size of EHS persons support groups and the experience of specialists. This means that *most people primed against EMF do not become EHS* – a fact that the authors apparently fail to consider.

Their commentary makes no mention of several situations, evoked in my review, where the predictions of their model do not materialize: the notable absence of EHS persons among activists against cell sites, who are obviously primed against EMF and aware of at least one source of exposure in their surroundings, and the existence of EHS persons that developed crippling symptoms years before learning about EMF or starting to suspect their environment. These observations indicate that the authors’ model could have a much narrower scope than they think, requiring very specific conditions to apply. Again, I think the authors are mistaken in avoiding this empirical discussion, and in disregarding any evidence that does not directly fit their view. This is not the way forward.

The conclusion I draw from this is the following. The authors’ model describes precisely how EHS symptoms *could* derive from, and reinforce, beliefs in EMF harmfulness. If EHS is to be understood, the most pressing question does not lie there anymore: it relates to *why* such beliefs really take hold in so few individuals, in which their presence is attested by significant alterations of lifestyle and behaviours. That question remains extremely difficult to address because very little is actually known of EHS persons, especially in relation to people suffering from other medically unexplained conditions or environmental intolerances. It shall thus become necessary to move away from experimental studies and the search for (psycho-)pathological mechanisms, at least temporarily, in favour of clinical and epidemiological studies using clear-cut definitions of EHS. This is what ultimately warrants the distinction between the cognitive and attributive hypotheses, no matter how conceptually misleading it appears to the authors: these hypotheses focus on different research questions requiring different empirical methods to be addressed. Should one of them be neglected, I fear that researchers will be condemned to endlessly reassert what is already known – while missing what is unknown – about EHS.

## Data Availability

not applicable.
